# GenFamClust: an accurate, synteny-aware and reliable homology inference algorithm

**DOI:** 10.1186/s12862-016-0684-2

**Published:** 2016-06-04

**Authors:** Raja H. Ali, Sayyed A. Muhammad, Lars Arvestad

**Affiliations:** KTH Royal Institute of Technology, Science for Life Laboratory, School of Computer Science and Communication, Solna, SE-171 77 Sweden; Department of Numerical Analysis and Computer Science, Stockholm University, Stockholm, SE-100 44 Sweden; Swedish e-Science Research Centre, Stockholm, Sweden; Science for Life Laboratory, Box 1031, Solna, SE-171 77 Sweden

**Keywords:** Homology inference, Gene synteny, Gene similarity, Gene family, Clustering, Gene order conservation

## Abstract

**Background:**

Homology inference is pivotal to evolutionary biology and is primarily based on significant sequence similarity, which, in general, is a good indicator of homology. Algorithms have also been designed to utilize conservation in gene order as an indication of homologous regions. We have developed GenFamClust, a method based on quantification of both gene order conservation and sequence similarity.

**Results:**

In this study, we validate GenFamClust by comparing it to well known homology inference algorithms on a synthetic dataset. We applied several popular clustering algorithms on homologs inferred by GenFamClust and other algorithms on a metazoan dataset and studied the outcomes. Accuracy, similarity, dependence, and other characteristics were investigated for gene families yielded by the clustering algorithms. GenFamClust was also applied to genes from a set of complete fungal genomes and gene families were inferred using clustering. The resulting gene families were compared with a manually curated gold standard of pillars from the Yeast Gene Order Browser. We found that the gene-order component of GenFamClust is simple, yet biologically realistic, and captures local synteny information for homologs.

**Conclusions:**

The study shows that GenFamClust is a more accurate, informed, and comprehensive pipeline to infer homologs and gene families than other commonly used homology and gene-family inference methods.

**Electronic supplementary material:**

The online version of this article (doi:10.1186/s12862-016-0684-2) contains supplementary material, which is available to authorized users.

## Background

Homology inference has been an active research topic of Computational Biology for several decades and is employed as a starting step for many studies in, for example, phylogeny inference, protein structure prediction [1], and function prediction [2, 3]. Our interest comes from the desire to define complete and suitable gene families for evolutionary studies on a genome-wide scale, i.e., when manual curation is out of the question, and homology inference is then the first step. Homology is mainly inferred from sequence similarity and there are issues that makes this seemingly easy problem inherently difficult (as the extensive literature on gene/protein homology inference is witness to). *Divergent families* can be hard to infer correctly due to weak similarity, even when combined with clustering methods, resulting in split families. Increasing search sensitivity to gain a larger number of homologous genes risks including non-homologs to gene families, for clustering methods based only on sequence similarity. *Convergent evolution*, causing similarity between two genes that do not have a common evolutionary origin [4], may not be the worst obstacle in general but can confuse inference [2]. Low-complexity regions are a further source of high local similarity between non-homologous proteins. Similarly, sequences of homologous proteins sometimes evolve at a fast rate and can not be identified by similarity-only based methods. *Multidomain proteins*, in particular those involving promiscuous domains [5], pose a special challenge for homology inference and have even been referred to as a problem ([6] for example). Shared domains are common and can link two proteins through a strong local similarity. Hence, multidomain proteins may be the main obstacle in homology inference and clustering as they break simplifying assumption on evolution. Inferring protein homology using local [7] or global [8] similarity depends on a model that does not take insertion of domains into account. This was recognized by Song et al. [9], who suggested a definition for “multidomain homology”. They stated that homologous proteins follow vertical inheritance and inserted domains (which are seen as horizontally transferred from another protein) should be discounted for. Explicitly identifying vertical inheritance is difficult, but they proposed a proxy based on a statistical analysis of conserved domain architecture.

Complementing a similarity-based method with additional information can improve analysis, as has been demonstrated for orthology inference, see [10], and we believe it valuable for homology inference as well. For orthologs, it is expected that genes’ locations are conserved at speciation, but rearrangements occur with time, so closely related species will display similar gene order. The same should also hold for many duplications. A chromosomal duplication retains gene order and although a duplication due to unequal crossing over disrupts gene order, copies in tandem retain almost the same neighborhood of genes. On the other hand, duplications due to retrotransposition often yield paralogs in new gene neighborhoods and reciprocal gene loss following large-scale duplications [11] break gene order. However, break in gene order for some duplications should not prevent us from making use of the conservation that is present and which provides a supportive signal for vertical inheritance. Signs of conserved gene order should boost the identification of potential homologs with weak similarity in general, and multidomain homologs in the sense of Song et al. [9] in particular. Hence, we wanted to investigate how conserved gene order may improve homology inference.

### A brief account of prior work

The primary, and recurring, tool for inferring homologs has been BLAST [12]. For example, BlastClust [13] uses single linkage clustering on BLAST results to compute clusters of homologs or gene families, and PSI-BLAST [12] uses an iterative procedure and position specific scoring matrix to infer remote homologs (homologous gene pairs with poor sequence identity but sharing common fold and function) from BLAST hits. Other gene family inference approaches are guided by multiple sequence alignment (MSA) likelihood (HiFiX [14]), profile hidden Markov models (Pfam [15]), and protein structure classification (SCOP [16]). hcluster_sg [17] is a graph-based algorithm that performs hierarchical agglomerative clustering on all-vs-all BLAST results. It is the clustering component of Ensembl Compara [18] and earlier versions of TreeFam [19]. Other algorithms have employed network structure and the transitive property of homology to infer gene families, e.g., Markov Clustering (MCL) [20], ProtoMap [21], TribeMCL [6], ProClust [22] and PHYRN (returns high resolution phylogenies alongwith distant gene families) [23].

An elegant solution to the multidomain homology problem, named Neighborhood Correlation (NC), has been proposed [9, 24]. NC avoids explicit identification of protein domains and architecture by looking at statistics of BLAST scores. It classifies two proteins as homologous if they have highly correlated BLAST scores when compared to a reference database, thereby avoiding the need for thresholds for alignment length, similarity, etc. The major advantage of NC over BLAST is its homology inference accuracy for diverse multidomain architecture proteins [25].

Algorithms have also combined other information with similarity to aid them in homology and/or orthology inference. Gene order conservation has been shown to be important information in homolog and ortholog validation as shown for fungi [26, 27] and prokaryotes [28, 29]. The SOAR and MSOAR algorithms have used synteny to assign orthologs by minimizing recombination distance between two genomes [30, 31]. Han and Hahn [32] have utilized local synteny information (conserved gene order within a fixed size neighborhood of queried pair) to identify parent-daughter relationships among duplicated genes. SYNERGY [33] uses gene order conservation (synteny) and species tree information alongside similarity to infer phylogeny and orthogroups, where an orthogroup for a specified node in the species tree is a set of genes that descended from or below the given node and is by definition a subset of homology. SYNS [34] and Jin et al. [35] use local synteny to infer homologs and orthologs.

In our earlier work [36], we presented a pipeline that builds on NC and combines its sequence similarity graph structure with gene order information to assess homology relationships. We showed that this approach, called GenFamClust (GFC), was more accurate than NC [36], which in turn has been shown to be better than BLAST scores [25].

### Present study

We performed a rudimentary accuracy check for homologs inferred from GFC and NC in our previous study [36] and NC has been compared with BLAST for accuracy [25]. Gene families inferred by applying clustering algorithms on homologs inferred from BLAST, NC or GFC have not been checked for other cluster properties. Therefore, we wanted to measure effects of different homology inference components on accuracy of gene family inference using selected clustering algorithms for simulated data and for a dataset with known gold standard. We also wanted to explore properties such as dependence, similarity, and latent class analysis between gene families. We wanted to quantify the effect of using synteny in addition with similarity for homology inference at the level of gene families using diverse biological datasets representing different branches in Tree of Life as well as on synthetic datasets.

GFC was evaluated in comparison with NC. We applied single, average and complete linkage clustering algorithms on homologs inferred from NC and from GFC, and applied MCL, hcluster_sg, SiLiX (a faster and memory-efficient implementation of BlastClust) [37] and HiFiX on BLAST scores to determine gene families. We calculated accuracy, similarity, dependence and other characteristics of gene families inferred from clustering algorithms on complete genomes of selected metazoan species, and validated the accuracy on synthetic datasets. We compared clusters inferred from applying clustering algorithms on homologs from GFC with semi-manually curated pillars, sets of orthologs and ohnologs (genes related by a whole genome duplication event) determined by Yeast Gene Order Browser (YGOB) [38] on complete genomes of a fungal dataset. We determined agreement and disagreement between GFC and YGOB. Our results suggest that GFC is a more reliable, accurate, informed and comprehensive pipeline to infer homology than most other similarity-based homology inference approaches as seen in diverse biological datasets as well as the synthetic datasets, and that the local synteny module of GFC adds biologically relevant and useful information to identify homologs.

## Methods

### The GFC method

**Fig. 1:**
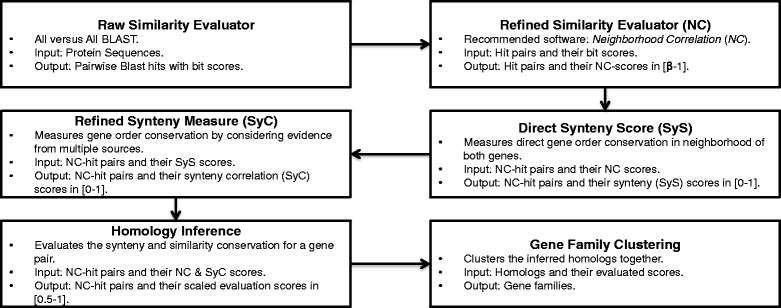
A brief introduction to GenFamClust that shows the modules and brief experimental settings of each module. The figure depicts the different modules, their functions, expected input, expected output and the author recommended software settings for each module

The GFC method [36] takes two (possibly intersecting) sets of sequences as input: query data *Q* and reference data *R*. *Q* should consist of genes for which we want to infer homology and *R* is only used when computing similarity and synteny correlation scores. In case we do not have reference data, the query data will be used as reference data. Figure 1 shows an overview of each module of GFC. GFC uses NC as its similarity measure and introduces a *synteny correlation score*, *SyC*, which in turn is inferred from a *local synteny score*, *SyS* as its synteny measure. We refer to the original work [36] for further details on parameter settings, method, comparison with NC, and other details.

In the previous study, we showed that GFC can work with data, where *Q* and *R* are disjoint and used query versus reference blast-scores as input. In the current study, input to GFC is all-versus-all BLAST scores. For two genes *g*_1_ and *g*_2_, the score *NC*(*g*_1_,*g*_2_) is (from [24])

$${{\begin{aligned} {}NC(g_{1},g_{2}) = \frac{\sum_{i\in N}(S(g_{1},i) - \overline{S}(g_{1}))(S(g_{2},i) - \overline{S}(g_{2}))} { \sqrt{\sum_{i\in N}(S(g_{1},i) - \overline{S}(g_{1}))^{2} \sum_{i\in N}(S(g_{2},i) - \overline{S}(g_{2}))^{2}}} \end{aligned}}} $$

where *S*(*g*_1_,*i*) is the normalized bit score of the optimal local alignment of query sequence *g*_1_ and database sequence *i*, *N* is the number of sequences in database, and $\overline {S}(g_{1})$ is mean of *S*(*g*_1_,*i*) over all sequences *i*.

The synteny scores *SyS*(*g*_1_,*g*_2_) between genes *g*_1_ and *g*_2_ are computed from NC scores. We define synteny score *SyS*(*g*_1_,*g*_2_) between two genes *g*_1_ and *g*_2_ as

$$SyS(g_{1},g_{2}) = \max\{NC(a,b) : a \in n(g_{1}), b \in n(g_{2})\} $$

where *n*(*g*) represents the set of neighbor genes, upstream or downstream of *g*, at most at distance *k*, on a chromosome or contig. In our previous study [36], we determined that *k*=5 is a suitable number of neighbouring genes upstream or downstream to consider for estimating local synteny between genes of Metazoa.

Synteny correlation score *SyC*(*g*_1_,*g*_2_) between genes *g*_1_ and *g*_2_ is defined as

$${{\begin{aligned} {}SyC(g_{1},g_{2}) \,=\, \frac{\sum_{i\in H}(SyS(g_{1},i) \,-\, \overline{SyS}(g_{1}))(SyS(g_{2},i) - \overline{SyS}(g_{2}))}{\sqrt{\sum_{i\in H}(SyS(g_{1},i)\! -\! \overline{SyS}(g_{1}))^{2} \sum_{i \in H}(SyS(g_{2},i) \,-\, \overline{SyS}(g_{2}))^{2}}} \end{aligned}}} $$

where ncHits(*g*_1_)={*i*|*i*∈*Q*∪*R*,*NC*(*g*_1_,*i*)≥*β*} and *H*=ncHits(*g*_1_)∩ncHits(*g*_2_).

Synteny correlation scores are calculated for each gene pair (*g*_1_, *g*_2_) with acceptable NC score, i.e., *g*_1_, *g*_2_∈*Q* and *NC*(*g*_1_,*g*_2_)>*β* (where *β* is a minimum threshold on NC score). Syntenic correlation score (SyC) is more robust than syntenic score (SyS) because SyS scores are negatively correlated to divergence times and conservation in gene order, and SyC is supported by evidence from a range of homologous regions from possibly multiple species with a range of divergence times. This gives empirical support to SyC scores as well as compensates for varying divergence times between species.

We use a heuristic decision boundary *h*(*g*_1_,*g*_2_) for a gene pair (*g*_1_, *g*_2_) as

$$h(g_{1}, g_{2}) = NC(g_{1}, g_{2})^{2} + 0.25 * SyC(g_{1}, g_{2})^{2} - 0.25 $$

where a positive value for *h*(*g*_1_,*g*_2_) indicates that *g*_1_ and *g*_2_ are homologous, otherwise *g*_1_ and *g*_2_ are classified as non-homologous. This decision boundary was determined and evaluated in the previous work [36] on the multispecies dataset and the specifics of evaluation are mentioned in the Additional file 1 of the same work.

### Data

For validating each homology inference method, we generated six synthetic datasets with known species tree, homology relationships, gene families and completely observable evolutionary history.

Two sets of biological data were collected comprising selected metazoan and fungal species. For these datasets, the genomes, proteomes, gene order information (gene to species mapping and their location on chromosomes), and the species tree are known. For genes with multiple protein isoforms (in particular from *Homo sapiens* and *Mus musculus*), we have selected the longest protein as a representative to maintain a single protein per gene representation. Both datasets have been taken from publicly available sources (Ensembl’s Metazoa genome browser and Yeast Gene Order Browser), and no experimentation was conducted on animals or humans.

#### Synthetic dataset

We generated the synthetic data using Artificial Life Framework (ALF) [39], which simulates major evolutionary forces at gene and genome level. We generated six datasets with varying synteny and similarity. *Mus musculus* chromosome 18 was used as ancestral chromosome due to its medium size of 497 genes and lower percentage of paralogs as compared to most other mouse and human chromosomes. Maximum indel size was set to 25, indel rate was set to 0.0005, and indel model was set to Zipfian distribution with distribution parameter *s* equal to 1.821. Duplication rates and loss rates were set so that total number of genes in each dataset was around 3000. For each simulation run, we varied substitution rate and translocation rate to alter evolutionary distance for similarity and synteny (Table 1). For simplicity, rates of all other evolutionary events, e.g., fusion, fission, neofunctionalization, etc., were set to zero. For further documentation on generation of simulated data, refer to Section 2.1 in Additional file 1.

**Table 1:** Parameter settings for generating the simulations using Artificial Life Framework (ALF)

Sim. #	# of families	Dup. rate	# of genes	Translocation rate	Subs. rate
1	329	0.0025	4272	0.002	350
2	289	0.0025	3837	0.001	350
3	382	0.0025	4065	0.0002	350
4	241	0.0045	4433	0.002	250
5	258	0.003	3941	0.001	250
6	233	0.003	3899	0.0002	250

Each dataset has a different substitution rate and/or translocation rate to show changes in gene sequence and order conservation. Datasets 1, 2 and 3 have lower sequence conservation while Datasets 4, 5 and 6 have higher sequence conservation. Similarly, Datasets 1 and 4 have low, 2 and 5 have medium and 3 and 6 have high gene order conservation

#### Metazoan dataset

The metazoan dataset consists of genomes from 19 species that range from primates and rodents, e.g., *Homo sapiens*, *Pongo abelii* and *Mus musculus* to simpler metazoans such as *Ciona intestinalis*. The genomes of this dataset have been extracted from Ensembl v. 72 [40] and the general properties and names of species present in this dataset are shown in Table 2. Model organisms representing major evolutionary branches in Metazoa with annotated and high quality genome assembly were selected from Ensembl’s Metazoa genome browser. The gold standard for this dataset consisted of 1561 genes divided into twenty function-based protein families from human and mouse taken from Song et al. [24]. These families were hand-curated and selected by a detailed literature study and other information such as domain and structure architecture. For each family, Song et al. [24] used Pfam and/or InterPro codes from publications by family experts, and reports from standards committees, such as the HUGO Gene Nomenclature Committee [41]. Table 3 enumerates basic properties of the gold standard dataset and further information regarding individual families and their characteristics can be found in Additional file 1: Section 2.3.

**Table 2:** Species in the metazoan dataset and their general properties

Scientific name	Common name	# scaffolds	# genes	genes/scaffold
*Takifugu rubripes*	Fugu	1,930	18,523	9.60
*Oreochromis niloticus*	Nile tilapia	1,081	21,437	19.83
*Danio rerio*	Zebra Fish	458	26,235	57.28
*Drosophila melanogaster*	Fruit fly	14	13,937	995.5
*Ciona intestinalis*	Sea squirt	732	16,658	22.76
*Loxodonta africana*	Elephant	583	20,033	34.36
*Tupaia belangeri*	Tree shrew	8,249	15,471	1.88
*Oryctolagus cuniculus*	Rabbit	1,022	19,018	18.61
*Sus scrofa*	Pig	1,367	21,607	15.81
*Equus caballus*	Horse	106	20,449	192.92
*Bos taurus*	Cow	43	19,994	464.98
*Homo sapiens*	Human	207	22,665	109.49
*Pongo abelii*	Orangutan	57	20,424	358.32
*Macaca mulatta*	Rhesus monkey	25	21,905	876.2
*Mus musculus*	House mouse	49	22,709	463.45
*Xenopus tropicalis*	Clawed frog	2,241	18,442	8.23
*Gallus gallus*	Chicken	809	15,508	19.17
*Taeniopygia guttata*	Zebra finch	70	17,488	249.83
*Anolis carolinensis*	Anolis lizard	2,425	18,596	7.67
Total	-	21,468	371,099	17.29

The table displays the common name, scientific name and the major GenBank taxonomic rank of species along with the gene and scaffold/chromosome distribution in each species present in metazoan dataset

**Table 3:** Properties of the “gold standard” metazoan dataset. This table enumerates the total number of positive and negative pairs on which cluster quality analysis is based

Description	No. of members
Sequences	1,561
Pairs	1,217,580
Families	20
Positive pairs	419,332
Negative pairs	798,248

#### Fungal dataset

**Fig. 2:**
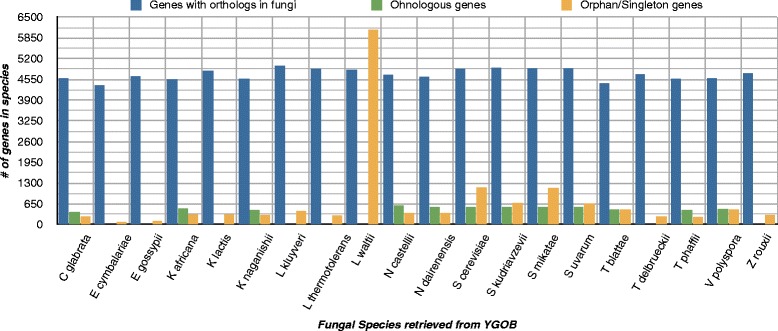
Species-wise distribution of genes in YGOB pillars. The distribution of genes for each species in YGOB v.7 pillars such that all genes that have an ohnolog or ortholog are quantitatively measured against all the singleton genes (genes that do not have an ohnolog or ortholog assigned) for each species. The distribution shows that *L. Waltii* has the most singleton genes

The Fungal dataset consists of well-assembled genomes from 20 Fungi species with a relatively conserved gene order, taken from Yeast Genome Order Browser (YGOB) version 7 [38] (also discussed in Section 2.2 of Additional file 1). YGOB partitions genes into sets related by either speciation or whole genome duplication events at their last common ancestor and terms such families as “pillars”. The YGOB database is composed of 12,596 pillars consisting of protein-coding genes. In YGOB, up to two genes from each species can be contained in a pillar. Pillars are delineated semi-automatically based on sequence similarity and synteny. We use pillars as a gold standard of orthology and ohnology (paralogous genes related by whole genome duplication). A whole genome duplication (WGD) in the Saccharomyces clade is hypothesized [26, 42–44] and YGOB classifies fungal species into pre-WGD species and post-WGD species in this context. To better understand distribution of genes, we classified genes in YGOB pillars into orthologs (at least one other gene in another species), singletons (no ortholog/ohnolog exists for these genes), and ohnologs (homologs related by WGD event), see Fig. 2.

#### Data grouping for GFC

For our synthetic data (all 6 datasets) and the fungal dataset, the complete datasets were used as query data *Q* for homology inference and as reference dataset *R*.

In the metazoan dataset, reference data *R* consisted of genomes from 18 species (shown in Table 2) and the query data *Q* was composed of *Homo sapiens* and *Mus musculus* genomes.

### Evaluation

#### Clustering strategies employed for BLAST, GFC and NC

The BLAST, NC, and GFC homology predictions were clustered using single linkage, complete linkage, and average linkage. SiLiX was used for inferring gene families using single-linkage clustering on BLAST. For computing average and complete linkage clusters with BLAST bitscores, normalized scores were used for clustering. For a pair of genes *g*_1_ and *g*_2_ with bitscore *s*(*g*_1_,*g*_2_), the normalized score is *s*(*g*_1_,*g*_2_)/ max(*s*(*g*_1_,*g*_1_),*s*(*g*_2_,*g*_2_)).

Names for clustering-based methods were constructed by appending the homology-inference method-name with the clustering strategy. For example, “GFC-Single” denotes that homology has been inferred using GFC followed by family inference using single linkage clustering.

#### Cluster quality analysis

To evaluate inferred clusters against known gene families (simulated with ALF or gold standard for biological datasets), we computed cluster quality *F*(*i*,*j*) for cluster *i* and family *j* using the F-score (also used by [25]), $F(i,j) = \frac {2P(i,j)R(i,j)}{P(i,j)+R(i,j)}$. This is the harmonic mean of precision *P*(*i*,*j*) (fraction of elements in cluster *j* that are members of family *i*) and recall *R*(*i*,*j*) (fraction of members of family *i* that are found in cluster *j*).

#### Evaluation on metazoan dataset

A precision-recall plot was drawn for all clustering methods on the metazoan dataset. One parameter from each software was identified as important for changing precision and recall, and this parameter was varied to change precision/recall. Note that the selected parameter was identified from a group of parameters by varying one parameter at a time on the metazoan dataset, and analysing the effect on precision and recall; other datasets may require a different parameter. Also, varying multiple parameters simultaneously might give a better precision and recall. Resulting clusters were then evaluated for precision and recall. Commands to run each software can be found in Additional file 1: Section 4. For hcluster_sg, applied on BLAST scores, no single parameter could be identified that caused significant variation for recall; settings used for Ensembl Compara were employed for hcluster_sg (i.e., maximum size 750, minimum edge weight 0 and minimum edge density between a join 0.34) and varied “minimum edge density between a join” parameter. We also used NC scores as input for hcluster_sg (this is referred to as NC-Hierarchical in further sections) and then identified “breaking edge density” as the parameter causing maximum variation. “Clustering threshold” was identified as the parameter for NC-based algorithms (NC and GFC) and for BLAST scores. The “inflation” parameter represented the most significant parameter for MCL and it was varied in the interval (1,5] (a range suggested by MCL authors [6]). The “MIN” parameter, representing minimum size of pre-families, in HiFiX was set to 10. SiLiX clusters produced in the analysis were given as input to vary HiFiX clusters.

#### Mutual information and Jaccard coefficient calculation

Mutual information represents dependence between two variables. Let *X* take values in {Yes,No} and denote the outcome from an inference method: homologous or not? Then the entropy of *X* is denoted as *H*(*X*) and calculated as *H*(*X*)= Pr(*X*=No) ln Pr(*X*=No)+ Pr(*X*=Yes) ln Pr(*X*=Yes). Given two different clustering methods *A* and *B*, the mutual information is *M*(*X*_*A*_,*X*_*B*_)=*H*(*X*_*A*_)+*H*(*X*_*B*_)−*H*(*X*_*A*_,*X*_*B*_), where *H*(*X*_*A*_,*X*_*B*_) is joint entropy and quantifies the overall agreement between both software *A* and *B*.

The Jaccard similarity coefficient is defined as *J*(*C*,*D*)=|*C*∩*D*|/|*C*∪*D*| where *C* and *D* are sets of inferred homologous pairs by two different methods.

#### Evaluation on the fungal dataset

The accuracy of GFC predictions on fungal dataset was ascertained by comparing clusters inferred from GFC homologs with YGOB pillars. We also looked for possible novel pillars, missed by YGOB but found as clusters of orthologs by GFC and evaluated them phylogenetically and syntenically. For phylogenetic analysis, we inferred gene trees from protein sequences of proposed gene family using a pipeline consisting of Clustal Omega [45] and FastTree [46] with default settings for both softwares. We used NOTUNG [47] with default settings to calculate the most parsimonious reconciliation (MPR, the reconciliation minimizing number of duplications) and to count and score the number of duplications and losses in MPR of inferred gene tree for gene cluster (minimum cluster size 4 since size 3 or less always have score 0) and the species tree [48]. NOTUNG’s D/L score is a weighted sum, where the default weights are 1.5 for duplications and 1.0 for losses. A lower score, or an increased cluster size with same score, indicated a better cluster because of implied closeness to MPR. This evaluation criteria is based on the hypothesis that a gene tree closer to MPR is more probable than the gene tree further from it [49, 50]. We also performed syntenic evaluation of both YGOB pillars and clusters inferred from GFC homologs, where we looked for synteny support for homologs. For syntenic analysis, we considered five neighboring genes upstream and five neighboring genes downstream of both genes and tried to find a BLAST hit in this neighborhood. If such a hit existed, then we concluded that the pair is syntenically supported.

## Results and discussion

We studied gene families inferred from GFC homologs for quality, agreement, recall, precision, and correlation with clusters from other software and available gold standards. The synthetic dataset was used to benchmark each software at several levels of similarity and synteny. We evaluated accuracy and statistical properties of clusters inferred from all software on metaozoan dataset. The fungal dataset was used to evaluate homology inference accuracy of GFC and importance of its syntenic measure.

### Synthetic dataset

#### Gene family quality analysis

**Fig. 3:**
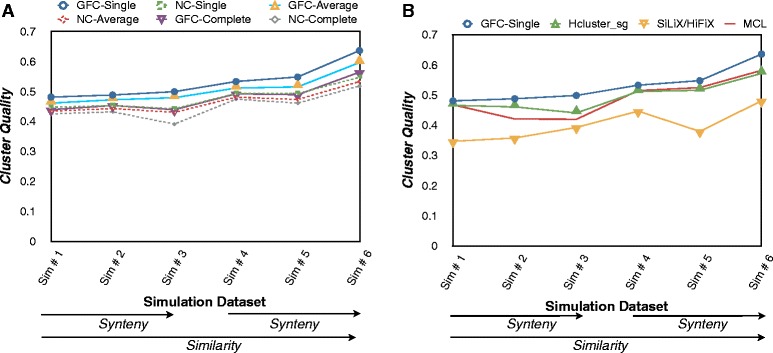
Cluster quality scores on simulated datasets for homology inference algorithms. The cluster quality scores of (**a**) single, average and complete linkage clustering when applied on homologs inferred by GenFamClust and by Neighborhood Correlation and (**b**) hcluster_sg, MCL, SiLiX and HiFiX clustering on BLAST scores and GenFamClust with single linkage clustering for each simulated dataset. Datasets are arranged in asscending order of similarity and then by asscending order of synteny. **a** Gene families inferred from GFC-based clustering methods (*solid lines*) are more accurate than those inferred from NC-based clustering methods (*dotted lines*) on all clustering algorithms and (**b**) Gene families inferred from GFC-Single (*blue line*) are more accurate than gene families inferred from similarity-only based clustering algorithms. The results are displayed in two panels for better legibility

We computed cluster quality *F*(*i*,*j*), precision *P*(*i*,*j*), and recall *R*(*i*,*j*) scores for all clustering methods on GFC and NC. As shown in Fig. 3a, GFC consistently performed better than NC for each clustering algorithm for all datasets with varying synteny and similarity conservation, indicating that synteny can improve inference. This is consistent with other studies [10, 34, 35], which shows that gene order conservation is extra information that can aid gene sequence conservation in inferring orthologs more accurately.

Similarly, we compared GFC-Single with other gene family inference methods. Hierarchical Clustering (hcluster_sg) and Markov clustering (MCL) performed consistently well as compared to NC-Single Linkage and outperformed SiLiX (using BlastClust-style single linkage clustering) and the MSA-based algorithm HiFiX. GFC-Single outperformed all these methods on clustering quality, see Fig. 3b. All clustering methods had a precision close to 1 for simulations 4, 5, and 6 (lower substitution rate) and 0.95 for the other three simulated datasets (higher substitution rate), but recall varied for different methods and datasets with GFC-Single having the best recall scores in all six datasets (data not shown). GFC-Single applied to datasets with high gene order conservation (simulations 2, 3, 5 and 6) unsurprisingly gave more accurate results than other homology inference methods. GFC-Single applied to the datasets with lowest gene order conservation (simulations 1 and 4) also showed considerable difference in gene cluster quality with other methods, see Fig. 3b.

### Metazoan dataset

#### Accuracy of gene family inference methods

In this section, we discuss the properties that can be derived from our biological datasets.

**Fig. 4:**
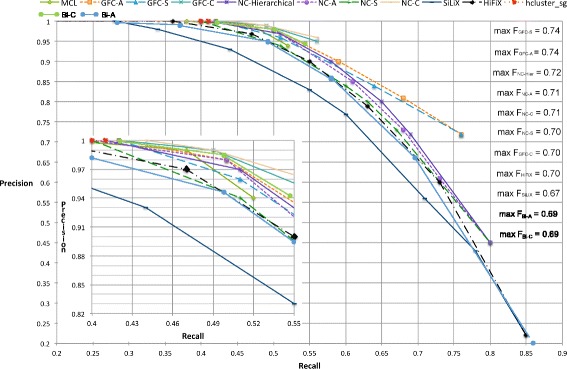
Precision-recall plot for precision and recall of various gene family inference methods on the metazoan dataset. The right top corner shows the maximum cluster quality for compared methods. GFC-Single and GFC-Complete have the best cluster quality followed by NC-Hierarchical and NC-Average. MCL, GFC-Complete and NC-Complete do not have data to test for recall beyond 0.54, 0.56 and 0.6 respectively. Other linkage algorithms on NC (NC-Single, NC-Average and NC-Hierarchical) have a maximum recall of 0.8 while GFC-Single and GFC-Average have a maximum recall of 0.75. Single linkage clustering on BLAST scores and HiFiX have maximum recall of 0.85. The inset zooms in for better legibility for recall between 0.45 and 0.6

We evaluated the accuracy of homologous pairs inferred from clusters by calculating precision and recall of all gene family inference methods. Figure 4 displays the receiver operating characteristic (ROC) curve for all gene family inference methods. While NC-Complete and GFC-Complete have a higher recall to precision ratio for the computed recall points, they have a smaller upper limit on recall. There are only two points for hcluster_sg because of the small variation we could achieve; the precision was close to 1.0, with a maximum recall of 0.41. The maximum recall observed for MCL is 0.52. Since NC is calculated for each BLAST hit, the maximum recall value of NC-based clustering methods is limited by the recall of BLAST-hit-based methods. GFC-based methods, in turn, are limited by NC, because GFC consider only those pairs (*g*_*i*_,*g*_*j*_) for homology evaluation that have *NC*(*g*_*i*_,*g*_*j*_)>0.3 (see *β* threshold in calculation of synteny correlation scores), but NC-based methods have a lower NC threshold of 0.05 for inferring homology.

Cluster quality scores for each method were also calculated, where the maximum cluster quality score for a method is shown in the top right of Fig. 4. GFC-Single and GFC-Average have the highest quality score followed by those of NC-Hierarchical and NC-Average. The maximum cluster quality-scores show small differences, which we interpret as a limitation with this indicator because there is substantial variation in the ROC curves. Synteny, domain architecture, and MSA likelihood provide additional information, on top of similarity scores, as shown by the area under the curve of clustering algorithms on GFC and on NC, and HiFiX as compared to clustering algorithms on BLAST scores alone (SiLiX and BLAST), as displayed in Fig. 4.

All methods except SiLiX have precision value 1 for recall values in [0–0.4]. NC-Complete, followed by GFC-Complete, have the highest precision values for recall values in [0.4–0.56]. GFC-Average, followed by NC-Average and NC-Hierarchical, has the highest precision to recall ratio for recall values in [0.56–0.75]. From the receiver operating characteristic (ROC) curve shown in Fig. 4, GFC-Average has a larger area under the curve. Also, GFC-Average and GFC-Single have the best cluster quality when maximum recall is desired. Therefore GFC-Average has the best precision to recall trade-off of all tested homology inference methods.

**Fig. 5:**
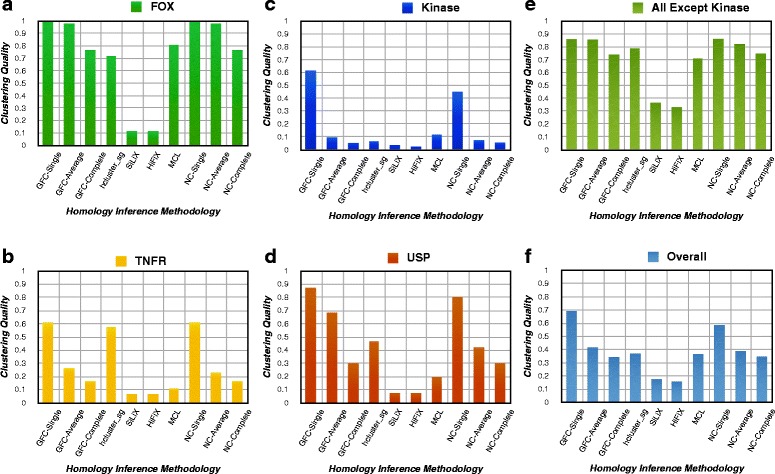
Cluster quality of various gene family inference methods on various families and the complete test data consisting of twenty families. The figure displays cluster quality of selected methods with default settings for various protein families as well as for twenty gene families in test data. Panels (**a**–**d**) display results for a particular protein family like FOX, TNFR, Kinase and USP respectively. FOX and TNFR are single domain architecture family while Kinase and USP have multidomain architecture with large sequence divergence and diverse domain architecture families. Panel (**e**) displays results for nineteen families (all except Kinases) because Kinases constitute more than half of proteins in number and could bias the overall results. Panel (**f**) displays the results for all twenty families. In all panels, GFC-based clustering methods have significantly higher or equal cluster quality than other methods

For a closer look at difficult families, we analyzed the 20 interesting gene families chosen by Song et al. [24] and computed their cluster quality, see Fig. 5 where panels a-d have data for the four example families FOX, TNFR, Kinase, and USP. GFC-Single and GFC-Average are as accurate or better than the corresponding algorithms of NC and significantly better than other methods in all cases. The difference can be clearly observed in Fig. 5a for the FOX family, where GFC-Single and NC-Single have quality score 1.0; GFC-Average and NC-Average have quality score 0.98 while all other algorithms are around or below 0.7. The same can also be noted for USP, a family with large sequence divergence, and the Kinase family, with rich variation in domain architecture. The significantly improved results of NC over other BLAST-based software (SiLiX and HiFiX) was described by Joseph et al. [25]. We have shown previously that the cluster quality of GFC is better than NC [36] and that is corroborated by results shown in Fig. 5 (experiments done on a different reference dataset). In particular Fig. 5e–f demonstrate overall improvement in cluster quality by GFC-Single and Average algorithms over other homology inference algorithms. The top three algorithms that are most accurate in inferring gene families for the overall test dataset are GFC Single (quality score 0.69), NC-Single (quality score 0.59) and GFC-Average (quality score 0.42) as displayed in Fig. 5f. However, Kinase is by far the largest family (900 members out of 1561 total members) and may bias the results towards methods performing well on this family. Therefore, we looked at what would happen if the Kinase members were excluded. The results retained the same trend, i.e., GFC-Single (quality score 0.86), NC-Single (quality score 0.86) and GFC-Average (quality score 0.85) are still the three best algorithms, see Fig. 5e.

#### Case studies on the value of synteny

To exemplify the value of synteny in assessing homology, we looked at two cases. The first case is an example of identifying multi-domain homology, because a domain insertion complicates similarity. The second case shows how high sequence divergence can be overcome thanks to neighboring genes.

**Fig. 6:**
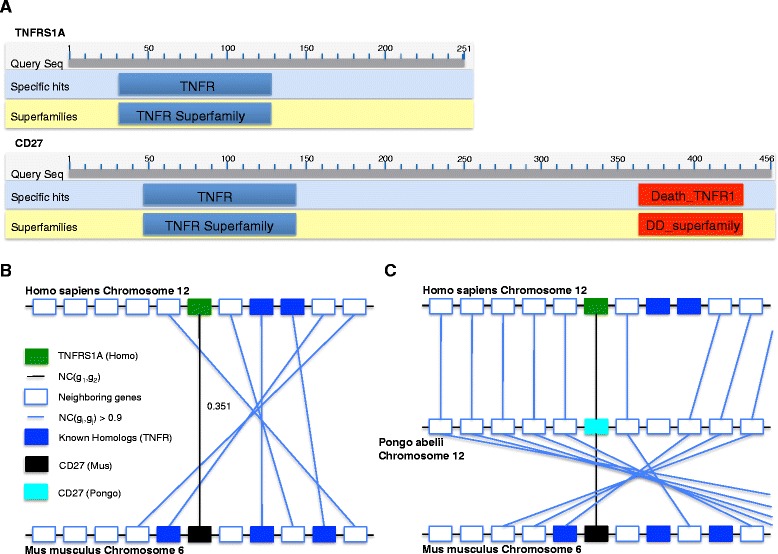
Case study: Local synteny adding useful information for homology inference for proteins with domain insertion. The genes CD27 in *Mus musculus* and TNFRSF1A in *Homo sapiens* are homologous and belong to the TNFR superfamily. **a** The domain architecture of CD27 and TNFRSF1A. CD27 has an additional Death_TNFR domain which is absent in TNFRSF1A. The NC-score (relative to the metazoan reference data) for CD27 and TNFRSF1A is 0.351, which is under the 0.5 threshold recommended for calling the genes homologous [9]. **b** and **c** Gene order conservation between *Homo sapiens* chr. 12, *Pongo abelii* chr. 12 and *Mus musculus* chr. 6 containing CD27 and TNFRSF1A at the center and five genes upstream and downstream in all three chromosomes. Only hits with NC-scores greater than 0.9 are displayed in these panels. Common NC-hits between CD27 gene in *Mus musculus* and TNFRSF1A gene in *Homo sapiens* are marked and used for calculating synteny correlation score between both genes. This is illustrated in (**c**) where CD27 in *Pongo abelii* is a common NC-hit of both genes. Direct synteny score between both genes (shown in **b**) using SyS score of GenFamClust is 0.991 and synteny correlation score between both genes using SyC score is 0.983. The GFC score obtained for this pair of genes is 0.115 which, being is greater than 0, indicates that CD27 and TNFRSF1A are homologs

TNFRSF1A in *Homo sapiens* and CD27 in *Mus musculus* are two genes belonging to the tumor necrosis factor receptor superfamily (TNFR), as classified by Song et al. [24] in the metazoan dataset. Figure 6a displays the domain architecture of both genes, where CD25 contains an additional Death_TRNF domain not present in TNFRSF1A. The BLAST bit-score between both genes is 43.5 and the E-value is 0.002. NC-score for the pair is 0.351, which is below the 0.50 threshold set for homologous gene pairs [25]. CD27 is found on chr. 6 in *Mus musculus* and TNFRSF1A on chr. 12 in *Homo sapiens*, very close to a cluster of identified members of TNFR superfamily. Figure 6b displays this region along with NC-hits (NC score >0.9) of genes within a distance of 5 genes upstream and downstream of CD27 and TNFRSF1A. Synteny score SyS(CD27, TNFRSF1A) is 0.991, which means that there is a very similar gene pair in the neighborhood of these genes. Synteny correlation score SyC(CD27, TNFRSF1A) is 0.983, suggesting conserved synteny in other species. Figure 6c displays the support for synteny for both CD27 and TNFRSF1A in *Pongo abelii* chr. 12, where both genes had a common NC-hit in *Pongo abelii* and this gene supports synteny scores of both genes. Thus when NC-score is combined with SyC scores for CD27 and TNFRSF1A, GFC infers these genes as a ln gene pair. Without support for synteny, NC would not be able to infer them as homologs with the current threshold.

**Fig. 7:**
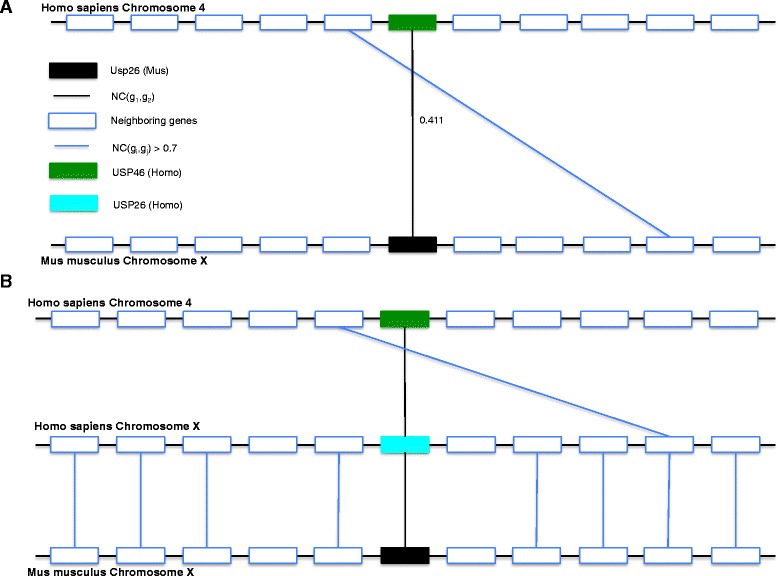
Case study: Local synteny adding useful information for homology inference for highly divergent proteins. The genes Usp26 in *Mus musculus* and USP46 in *Homo sapiens* are homologous and belong to the USP superfamily. Both proteins are single domain proteins belonging to the Peptidase_C19 superfamily. The NC-score (relative to the metazoan reference data) for USP46 and Usp26 is 0.411, which is under the 0.5 threshold recommended for calling the genes homologous [9]. **a** and **b** Gene order conservation between *Homo sapiens* chr. 4, *Homo sapiens* chr. X and *Mus musculus* chr. X containing Usp26 and USP46 at the center and five genes upstream and downstream in all three chromosomes. Only hits with NC-scores greater than 0.7 are displayed in these panels. Common NC-hits between Usp26 gene in *Mus musculus* and USP46 gene in *Homo sapiens* are marked and used for calculating synteny correlation score between both genes. This is illustrated in (**b**) where USP26 in *Homo sapiens* is a common NC-hit of both genes. Direct synteny score between both genes (shown in **a**) using SyS score of GenFamClust is 0.741 and synteny correlation score between both genes using SyC score is 0.672. The GFC positive score obtained for this pair of genes, 0.045, indicates that Usp26 and USP46 are homologs despite high sequence divergence and little sequence similarity

In the second case study, USP46 in *Homo sapiens* and Usp26 in *Mus musculus*, belonging to the ubiquitin specific peptidase (USP) superfamily, are two homologous genes in the metazoan dataset. Both proteins are single domain proteins containing the Peptidase_C19 domain. The BLAST bit-score for the two proteins is 48.1 and their E-value is 0.0006. Their NC-score is 0.411, which is below the 0.50 threshold suggested for homologous gene pairs [25]. USP46 is found on chr. 4 in *Homo sapiens* and Usp26 on chr. X in *Mus musculus* with significant sequence divergence between the two proteins. Figure 7a displays this region along with NC-hits (NC score >0.7) of genes within a distance of 5 genes upstream and downstream of USP46 and Usp26. Synteny score SyS(USP46, Usp26) is 0.741 due to presence of a similar gene pair (SPETEX1 in Homo sapiens and Ccdc160 in Mus musculus) in the neighborhood of these genes. Synteny correlation score SyC(USP46, Usp26) is 0.672, suggesting conserved synteny in other species. Figure 7b displays the support for synteny for both USP46 and Usp26 in *Homo sapiens* chr. X, where both genes have a common NC-hit (USP26) in *Homo sapiens* and syntenic conservation of both query genes is supported by this region. Thus, when NC-score is combined with SyC scores, GFC infers these genes as a homologous gene pair. Due to high sequence divergence and lack of sequence similarity, NC and BLAST do not infer them as homologs with common thresholds.

#### Similarity and statistical dependence between gene family inference methods

The similarities and dependence between various gene family inference methods was assessed by calculating Jaccard similarity coefficients and mutual information scores for all pairwise comparisons for the methods. Mutual information (Table 4, top) reflects the overall dependence and is used to assess correlation between two variables (methods in this case). All methods are most correlated with GFC-Single (top eight are highlighted), which supports the proposition that GFC-Single is a good replacement for other methods. However, when Jaccard similarity coefficients (which reflect the ratio between number of genes both methods assign to the same cluster and the number of decisions in which at least one software assigns a particular gene to this cluster between both software) were calculated for the pairwise data (Table 4, bottom), the top eight values (shown in bold-face) showed that GFC-Single does not have a single top most value. In fact, the best values belong to GFC-Average and GFC-Complete, which suggests that other methods have little disagreement on clustering of genes with GFC-Average and GFC-Complete.

**Table 4:** Similarity and statistical dependence between homology inference methods. Mutual information scores are found above the diagonal and Jaccard similarity coefficient are found below the diagonal

Mutual information/		GenFamClust			Neighborhood correlation			SiLiX	MCL	hcluster_sg
Jaccard coefficient		Aver	Comp	Sing	Aver	Comp	Sing			
**GFC**	**Average**		0.146	**0.628**	0.139	0.145	0.517	0.149	0.167	0.159
	**Complete**	0.599		**0.604**	0.123	0.094	0.497	0.112	0.158	0.125
	**Single**	0.117	0.071		**0.618**	**0.605**	**0.785**	**0.581**	**0.631**	**0.613**
**NC**	**Average**	**0.815**	**0.725**	0.096		0.121	0.508	0.130	0.164	0.143
	**Complete**	0.621	**0.925**	0.073	**0.758**		0.498	0.113	0.158	0.125
	**Single**	0.169	0.103	0.573	0.138	0.106		0.477	0.518	0.505
**SiLiX**		0.273	0.339	0.037	0.320	0.345	0.053		0.154	0.126
**MCL**		**0.733**	0.520	0.122	**0.647**	0.537	0.176	0.261		0.173
**hcluster_sg**		0.586	**0.646**	0.083	0.623	**0.658**	0.119	0.277	0.496	

The eight highest values for Jacquard’s similarity coefficient (below the diagonal) and mutual information (above the diagonal) are bold-faced to show the pair of software with most similarity and statistical dependence

#### Method agreement

**Fig. 8:**
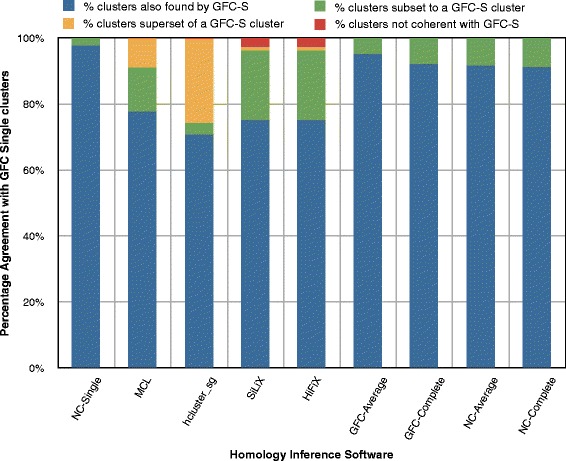
Cluster coherence for GFC-Single with other gene family inference methods. The bar chart displays coherence at cluster level between GFC-Single and other gene family inference methods. A cluster is termed common cluster if it can be found with exactly the same members in both softwares. A cluster is termed as subset if the first software contains two or more clusters merged together as a single cluster in the second software. Any cluster which is neither common nor subset (or superset) is considered contradictory. As expected, NC-based methods, i.e., NC-Single, NC-Average, NC-Complete, GFC-Average and GFC-Complete have the most number of clusters in common with GFC-Single (shown with blue parts of the bar) and there are no contradictory clusters between these software and GFC-Single. However, other software (HiFiX, MCL, hcluster_sg and SiLiX) have relatively less common clusters with GFC-Single and a few contradictory clusters can also be observed for HiFiX and SiLiX

We investigated the clusters formed by GFC-Single matching for coherence with other methods, i.e., where two clusters A and B are coherent if *A*⊂*B* or *A*⊃*B* or *A*=*B*. Two clusters are not coherent if their intersection is not empty and both contain at least one member not found in the other cluster. Figure 8 shows the percentage of clusters inferred by GFC-Single that are coherent with other homology inference method. Since NC is a component of GFC and average and complete linkage are more strict algorithms than single linkage, it is not surprising that GFC-Single is 100 % coherent with all clustering algorithms on NC and average and complete linkage clustering on GFC. Unsurprisingly, all clusters in GFC-Single are either superset or equal to the clusters formed by these clustering methods due to the high agreement between GFC-Single and other methods. Furthermore, as expected from high values in the mutual information scores (Table 4), more than 95 % of GFC-Single clusters are coherent when comparing with all other methods.

**Fig. 9:**
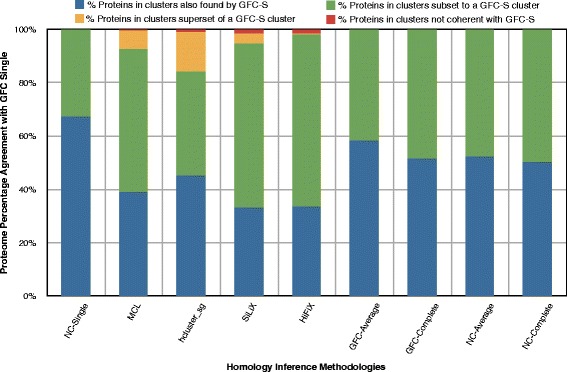
Protein distribution by GFC-Single in comparison with other gene family inference methods. Bar chart displays the protein distribution according to the cluster a protein is found in, where clusters can be common, subset, superset or contradictory as discussed before. The protein distribution in each class shows aggressive clustering behavior of GFC-based clustering methods w.r.t. other software, i.e., when compared with the corresponding bar in Fig. 8, we notice that the percentage proteins contained in common clusters is significantly less than the percentage of common clusters while the percentage proteins contained in subset clusters of GFC-Single has substantially increased than the percentage of subset clusters of GFC-Single for all software

The cluster size (number of proteins in each cluster) is an important characteristic of gene families. A method that aims to find the maximum number of true homologs may have a high false positive rate and gives large-sized clusters. On the other hand, a more precise method will trade off on the number of true homology relationships it captures and might find relatively small and average sized clusters. In order to study cluster sizes and see effect of each clustering strategy, we counted the number of proteins (shown in Fig. 9) in each coherent sub-group for each method, with respect to GFC-Single, see Fig. 8. GFC-Single and SiLiX form a few large clusters reflected in Fig. 8 and in Fig. 9 for each corresponding method (2.42 % clusters from GFC-Single correspond to 32.68 % proteins clustered by GFC-Single for NC-Single displayed by green part of first bar in both Fig. 8 and in Fig. 9 and similarly for all other methods) and similarly for SiLiX (0.4 % clusters of GFC-Single corresponds to 7 % proteins clustered by SiLiX). On the other hand, clusters formed by GFC-Average are similar to most other software (data not shown here).

One interesting observation is the behavior of hcluster_sg, where a large proportion of GFC-Single clusters are subsets of hcluster_sg clusters (25.35 %), yet contains only 14.76 % of proteins clustered by GFC-Single (shown by yellow bars for hcluster_sg in Fig. 8 and in Fig. 9). This is because of the compactness constraint imposed by the hcluster_sg algorithm. A similar pattern is also observed for clusters formed by MCL with respect to clusters by GFC-Single.

### Quality assessment using the fungal dataset

#### Accuracy of gene family inference of GenFamClust

We wanted to measure the accuracy of gene family inference of GFC with a semi-automatic curated gold standard on a dataset with good syntenic support. The pipeline employed by YGOB uses an automatic clustering (where each cluster/gene family is referred to as pillars) based on BLAST scores and synteny, followed by a round of manual curation [38]. Some additional genes are assigned based on indirect evidence, via a mutual homolog or by a combination of other observations: synteny in the other post-WGD genomes (genomes that underwent a whole genome duplication event) and pre-WGD genomes (genomes that did not undergo whole genome duplication) at that locus, two copies of the gene in other post-WGD genomes, correct clades in phylogenetic trees, or similar protein lengths [38]. Thanks to the manual curation, YGOB can be used to benchmark clusters/gene families formed by GFC. Note that there is relatively strong synteny in yeasts, so the synteny-support used by YGOB is not as generally applicable for other datasets as it is for yeasts whereas the synteny quantification method of GFC can be used for datasets with various degrees of syntenic conservation.

**Fig. 10:**
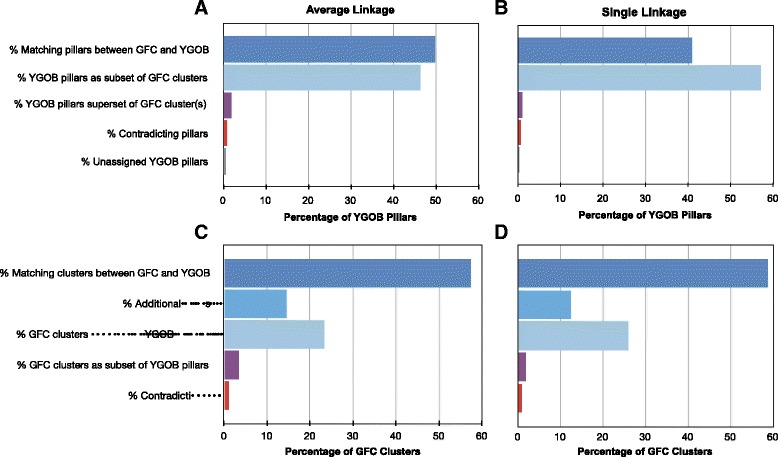
GFC cluster agreement with YGOB pillars. The figure shows bar charts displaying the agreement and disagreement between YGOB pillars and clusters formed by single linkage and average linkage on homologs inferred from GFC. First, clusters determined by GFC are mapped onto YGOB pillars in (**a**) for GFC-Average and in (**b**) for GFC-Single, where each bar displays the percentage of YGOB pillars in that category. Second, YGOB pillars are mapped onto clusters inferred by GFC-Average in (**c**) and in (**d**) by GFC-Single, where each bar represents percentage of GFC clusters in that category. The track “additional pillars” displays the percentage of clusters looking like a pillar, with genes are from different species and containing singleton genes in YGOB. The track “GFC clusters superset of YGOB pillars” represents the percentage of GFC clusters that contain one or more YGOB pillar(s) completely. The track “contradictions” represents the percentage of YGOB pillars/GFC clusters where GFC places two genes from same YGOB pillar in different clusters

The YGOB pillars contain only orthologs and ohnologs, while GFC clusters infer homologs that also contain paralogs. Hence, relating clusters and pillars, a successful cluster is either identical to, or a superset of, a YGOB pillar. We first mapped GFC clusters to the YGOB pillars, see Fig. 10a–b, and looked at both single linkage and average linkage clustering with GFC. More than 96 % of pillars in YGOB are coherent with both single and average linkage clusters from GFC. Only 2.7 % of the pillars are broken up into smaller clusters by GFC-Average and 1.3 % by GFC-Single. There is disagreement for 1.0 % of pillars with GFC-Average and for 0.6 % with GFC-Single. Therefore, 3.7 % of YGOB pillars disagree with GFC-Average clusters and 1.9 % of them with GFC-Single clusters. This illustrates that most of YGOB pillars are also inferred by GFC-Single and GFC-Average clustering.

We wanted to see if clusters inferred from GFC can be predicted as “novel pillars”, i.e., pillars that YGOB has missed. For this purpose, we mapped YGOB pillars onto GFC-Single or GFC-Average clusters and classified clusters as coherent, contradictory and putative novel pillars as shown in Fig. 10c–d. Clusters from GFC-Average or GFC-Single containing complete YGOB pillars are termed as coherent. If a cluster of GFC-Single or GFC-Average cluster contains an incomplete YGOB pillar, then this cluster is classified as contradictory. Putative novel pillars consist of those genes that exist as singleton genes or in two or more disjoint pillars in YGOB dataset, but are brought together by GFC-Single/GFC-Average, forming a multi-gene or a multi-pillar cluster. 14.6 % of GFC-Average clusters are putative novel pillars while 12.5 % of GFC-Single clusters are putative novel pillars. Most of the genes in these putative pillars are paralogs (two or more genes for at least one species), but a few clusters can be characterized as pillars based on their one-to-one species correspondence.

#### Analysis of novel and contradictory predictions of GenFamClust

We further investigated clusters from GFC-Single or GFC-Average that contradicted YGOB pillars, i.e., those clusters that contain at least one partial YGOB pillar. We analyzed gene order support for both YGOB pillars and GFC Single or GFC-Average clusters by looking for a similarity hit in neighborhood of all gene pairs in cluster. Since GFC uses a synteny correlation score explicitly in homology inference and YGOB also uses synteny for pillar inference, both GFC-Single or GFC-Average clusters and YGOB pillars show good syntenic support (89.0 % for GFC clusters and 91.2 % for YGOB pillars contain a homologous gene pair within 5 neighboring genes). It is through phylogenetic analysis that the differences between YGOB pillars and clusters of GFC-Single or GFC-Average are highlighted. We inferred phylogenetic trees and computed gene/species tree reconciliations with associated D/L scores (from NOTUNG) for clusters and pillars, see Table 5. All clusters/pillars with size < 4 were excluded as they always have a D/L score of 0. For all three cases, i.e., novel pillars proposed by GFC, pillars contained within GFC and GFC clusters contained within YGOB pillars, the average D/L score per gene of clusters from GFC-Single or GFC-Average is significantly lower than that of YGOB pillars implying that on average, each cluster from GFC-Single or GFC-Average is phylogenetically more parsimonious than the corresponding YGOB pillar. Our manual inspection of the data suggests that GFC finds pillars with high sequence divergence that are mistakenly split up by YGOB. Manual curation is supposed to find and correct such cases in YGOB, but the automated use of synteny in GFC seems more successful.

**Table 5:** Phylogenetic analysis of all contradicting GFC cluster and YGOB pillars

Pillars	Method	Dupl’s	Losses	D/L scores	# genes	Score per gene
YGOB pillars not found in GFC	YGOB	53	219	298.5	196	1.523
	GFC	-	-	-	-	-
GFC clusters containing probable pillars	YGOB	-	-	-	-	-
	GFC	68	269	371	306	1.212
YGOB pillars containing GFC clusters	YGOB	438	1837	2494	1321	1.888
	GFC	367	1448	1998.5	1221	1.637
GFC clusters containing YGOB pillars	YGOB	43	164	228.5	126	1.813
	GFC	58	210	297	188	1.580

The phylogenetic quality of clusters formed by GFC-Average linkage clusters and alternatives suggested by YGOB is shown here. The pillars/clusters are of size ≥ 4 and includes pillars not clustered together by GFC and vice versa, GFC clusters contained in YGOB and probable pillar-like clusters (at most one member from each pre-WGD species and at most two members from each post-WGD species with at least one ortholog) that contain YGOB clusters. The numbers of genes in each cluster are summed up to find the total number of genes. NOTUNG’s D/L score divided by the total number of genes gives the average score per gene, where a lower ratio indicates a better fit according to the MPR

**Fig. 11:**
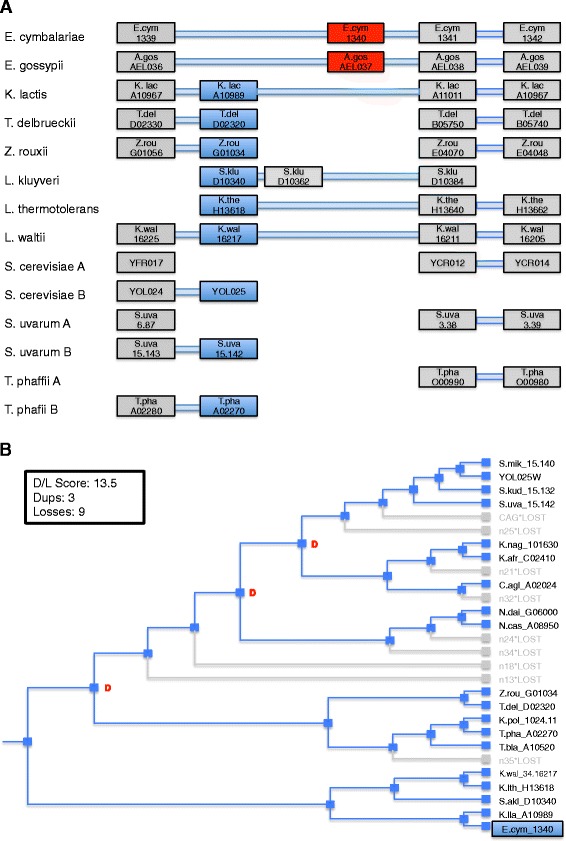
Case study: Difference between GFC cluster and YGOB pillars - Phylogenetic analysis. **a** displays a portion of 6 YGOB pillars, where we are interested in the two pillars (*red* and *blue* columns). YGOB classifies them as separate clusters as shown in (**a**) but GFC-Single and GFC-Average classifies Ecym_1340 to be part of *blue pillar* and AEL037C to be a singleton gene. **a** also shows that both the cluster and the pillar has good syntenic support via neighboring pillars. **b** displays the D/L score and the most parsimonious reconciliation of gene tree with species tree constructed from GFC cluster with/without Ecym_1340 added and highlighted here in *blue*. The cluster given by GFC-based methods (Ecym_1340 added to the *blue cluster* in **a**) has the lowest D/L score to gene ratio and is, therefore, phylogenetically most probable. There exist multiple genes in the *blue pillar* that have a BLAST hit with Ecym_1340 and there does not exist a BLAST hit with evalue 10 between Ecym_1340 and AEL037C showing similarity support for GFC cluster and lack of similarity for the genes in *red pillar* in (**a**)

In Fig. 11, we present a case where a cluster determined by GFC-Average and its corresponding YGOB pillar do not agree. In Fig. 11a, each column represents a YGOB pillar and the specific pillars concerning this case are differentiated from other pillars by using red and dark blue colors. YGOB places E.cym_1340 (*E. cymbalariae*) along with AEL037C (*E. gossypii*) as displayed in Fig. 11a (pillar shown with red). However, GFC-Average places E.cym_1340 in the pillar containing TDEL0D02320 (*T. delbrueckii*) and KLLA0A10989g (*K. lactis*) (pillar shown by dark blue) and shows AEL037C as a single gene cluster. While both placements of Ecym_1340 are syntenically supported, there are a couple of drawbacks with placing it alongside AEL037C. First, Ecym_1340 has weak BLAST hits with most of the members of the blue pillar in Fig. 11a but no BLAST hit with AEL037C within E-value of 10. Therefore placing Ecym_1340 within the pillar containing TDEL0D02320 is better choice on account of similarity with other members of the pillar than placing with AEL037C. Second, as shown in Fig. 11b (same number of duplications and losses with/without adding Ecym_1340 to the blue cluster) phylogenetic analysis reveals that GFC cluster easily accommodates Ecym_1340 without adding any duplication or loss to the reconcialiation, which means that the GFC cluster has lower duplication and loss score per gene than the corresponding YGOB pillar. Therefore, in this case, both phylogenetically and through sequence similarity analysis, it is concluded that the cluster inferred on GFC-homologs is more plausible than the YGOB pillar.

## Conclusions

Homology and gene family inference are important tasks and prerequisites to functional, structural, and phylogenetic characterization of genes and proteins. Multidomain proteins constitute a long-standing challenge, both in terms of interpretation and prediction of homology. Based on the Neighborhood Correlation framework [24, 25], we have in previous work suggested a novel method, GenFamClust that makes use of network structure of synteny and similarity across multiple genomes [36]. Here, we have evaluated GenFamClust in comparison with other homology inference like Neighborhood Correlation and BLAST using clustering approaches like single linkage, complete linkage, average linkage, Markov Clustering, hcluster_sg, SiLiX and HiFix on accuracy, similarity, dependence and/or other characteristics on complete genomes of metazoan dataset and validating accuracy of all software on simulated datasets. Clustering algorithms applied on GenFamClust show maximum similarity with other gene family inference methods using mutual information and Jaccard similarity coefficient. Moreover, we compared performance of GenFamClust on complete genomes of a fungal dataset with semi-manually curated orthologs and ohnologs (genes related by a whole genome duplication event) to determine accuracy of GenFamClust and show that the clusters, where GenFamClust and Yeast Gene Order Browser pillars disagree, the ones formed by GenFamClust have on average a lower duplication loss score per gene, are on average closer to the most parsimonious reconciliation with the fungal species tree and therefore more accurate than the pillars. Furthermore, quantitative synteny is a useful way of incorporating gene order conservation information in homology inference. There was significant improvement in results, particularly for datasets containing homologs with syntenic support (e.g., orthologs, and paralogs related by segmental duplication), despite GenFamClust’s relatively simple approach to assessing gene order. From this work, we conclude that GenFamClust is a more accurate method to infer homology than most other common homology inference approaches and its synteny measure is simple and biologically realistic as seen in diverse biological datasets as well as in simulated datasets. Importantly, it is a tool that uses both similarity and synteny to explicitly predict multidomain homology.

## Abbreviations

ALF: artificial life framework; BLAST: basic local alignment search tool; D/L: duplication/loss; GFC: genfamclust; LCA: last common ancestor; MCL: Markov clustering; MPR: most parsimonious reconciliation; MSA: multiple sequence alignment; NC: neighborhood correlation; PSI-BLAST: position-specific iterative basic local alignment search tool; ROC: receiver operating characteristic; TNFR: tumor necrosis factor receptor; USP: ubiquitin specific peptidase; WGD: whole genome duplication; YGOB: yeast gene order browser

## Additional file

Additional file 1 Additional documentation. Supplementary file containing additional documentation, source and processing of data sets, commands used to execute different software, and how the figures and tables were generated from raw data. (PDF 137 kb)

## References

[1] Levitt M (2001). The birth of computational structural biology. Nat Struct Biol.

[2] Fitch WM (1970). Distinguishing homologous from analogous proteins. Syst Zool.

[3] Koonin EV (2005). Orthologs, paralogs, and evolutionary genomics. Annu Rev Genet.

[4] Parker J, Tsagkogeorga G, Cotton JA, Liu Y, Provero P, Stupka E, Rossiter SJ (2013). Genome-wide signatures of convergent evolution in echolocating mammals. Nature.

[5] Basu MK, Carmel L, Rogozin IB, Koonin EV (2008). Evolution of protein domain promiscuity in eukaryotes. Genome Res.

[6] Enright AJ, van Dongen S, Ouzounis CA (2002). An efficient algorithm for large-scale detection of protein families. Nucleic Acids Res.

[7] Smith TF, Waterman MS (1981). Identification of common molecular subsequences. J Mol Biol.

[8] Needleman SB, Wunsch CD (1970). A general method applicable to the search for similarities in the amino acid sequence of two proteins. J Mol Biol.

[9] Song N, Sedgewick RD, Durand D (2007). Domain architecture comparison for multidomain homology identification. J Comput Biol.

[10] Kristensen D, Wolf Y, Mushegian A, Koonin E (2011). Computational methods for gene orthology inference. Brief Bioinform.

[11] Scannell DR, Byrne KP, Gordon JL, Wong S, Wolfe KH (2006). Multiple rounds of speciation associated with reciprocal gene loss in polyploid yeasts. Nature.

[12] Altschul SF, Madden TL, Schäffer AA, Zhang J, Zhang Z, Miller W, Lipman DJ (1997). Gapped BLAST and PSI-BLAST: a new generation of protein database search programs. Nucleic Acids Res.

[13] NCBI. Using BLASTClust to make non-redundant sequence sets. ftp://ftp.ncbi.nih.gov/blast/documents/blastclust.html. Accessed 19 May 2016.

[14] Miele V, Penel S, Daubin V, Picard F, Kahn D, Duret L (2012). High-quality sequence clustering guided by network topology and multiple alignment likelihood. Bioinformatics.

[15] Finn RD, Bateman A, Clements J, Coggill P, Eberhardt RY, Eddy SR, Heger A, Hetherington K, Holm L, Mistry J, Sonnhammer ELL, Tate J, Punta M (2014). Pfam: the protein families database. Nucleic Acids Res.

[16] Conte LL, Ailey B, Hubbard TJP, Brenner SE, Murzin AG, Chothia C (2000). SCOP: a structural classification of proteins database. Nucleic Acids Res.

[17] Li H. Constructing the TreeFam database. PhD thesis, Chinese Academy of Sciences Beijing. 2006. http://lh3lh3.users.sourceforge.net/download/PhD-thesis-liheng-2006-English.pdf. Accessed 19 May 2016.

[18] Vilella AJ, Severin J, Ureta-Vidal A, Heng L, Durbin R, Birney E (2009). EnsemblCompara GeneTrees: Complete, duplication-aware phylogenetic trees in vertebrates. Genome Res.

[19] Li H, Coghlan A, Ruan J, Coin LJ, Hériché JK, Osmotherly L, Li R, Liu T, Zhang Z, Bolund L, Wong GK, Zheng W, Dehal P, Wang J, Durbin R (2005). TreeFam: a curated database of phylogenetic trees of animal gene families. Nucleic Acids Res.

[20] van Dongen S. Graph clustering by flow simulation. PhD thesis, University of Utrecht Netherlands. 2000. http://micans.org/mcl/lit/svdthesis.pdf.gz. Accessed 19 May 2016.

[21] Yona G, Linial N, Linial M (2000). ProtoMap: automatic classification of protein sequences and hierarchy of protein families. Nucleic Acids Res.

[22] Pipenbacher P, Schliep A, Schneckener S, Schönhuth A, Schomburg D, Schrader R (2002). ProClust: improved clustering of protein sequences with an extended graph-based approach. Bioinformatics (Oxford, England).

[23] Bhardwaj G, Ko KD, Hong Y, Zhang Z, Ho NL, Chintapalli SV, Kline LA, Gotlin M, Hartranft DN, Patterson ME, Dave F, Smith EJ, Holmes EC, Patterson RL, van Rossum DB (2012). PHYRN: a robust method for phylogenetic analysis of highly divergent sequences. PloS One.

[24] Song N, Joseph JM, Davis GB, Durand D (2008). Sequence similarity network reveals common ancestry of multidomain proteins. PLoS Comput Biol.

[25] Joseph JM, Durand D (2009). Family classification without domain chaining. Bioinformatics.

[26] Kellis M, Birren BW, Lander ES (2004). Proof and evolutionary analysis of ancient genome duplication in the yeast Saccharomyces cerevisiae. Nature.

[27] Wapinski I, Pfeffer A, Friedman N, Regev A (2007). Natural history and evolutionary principles of gene duplication in fungi. Nature.

[28] Lemoine F, Lespinet O, Labedan B (2007). Assessing the evolutionary rate of positional orthologous genes in prokaryotes using synteny data. BMC Evol Biol.

[29] Lemoine F, Labedan B, Lespinet O (2008). SynteBase/SynteView: a tool to visualize gene order conservation in prokaryotic genomes. BMC Bioinformatics.

[30] Fu Z, Chen X, Vacic V, Nan P, Zhong Y, Jiang T, Apostolico A, Guerra C, Istrail S, Pevzner PA, Waterman M (2006). A parsimony approach to genome-wide ortholog assignment. Research in Computational Molecular Biology: 10th Annual International Conference, RECOMB 2006, Venice, Italy, 2006. Proceedings.

[31] Fu Z, Chen X, Vacic V, Nan P, Zhong Y, Jiang T (2007). MSOAR: a high-throughput ortholog assignment system based on genome rearrangement. J Comput Biol.

[32] Han MV, Hahn MW (2009). Identifying parent-daughter relationships among duplicated genes. Pac Symp Biocomput.

[33] Wapinski I, Pfeffer A, Friedman N, Regev A (2007). Automatic genome-wide reconstruction of phylogenetic gene trees. Bioinformatics.

[34] Sarkar A, Soueidan H, Nikolski M (2011). Identification of conserved gene clusters in multiple genomes based on synteny and homology. BMC Bioinformatics.

[35] Jun J, Mandoiu II, Nelson CE (2009). Identification of mammalian orthologs using local synteny. BMC Genomics.

[36] Ali RH, Muhammad SA, Khan MA, Arvestad L (2013). Quantitative synteny scoring improves homology inference and partitioning of gene families. BMC Bioinforma.

[37] Miele V, Penel S, Duret L (2011). Ultra-fast sequence clustering from similarity networks with SiLiX. BMC Bioinforma.

[38] Byrne KP, Wolfe KH (2005). The Yeast Gene Order Browser: combining curated homology and syntenic context reveals gene fate in polyploid species. Genome Res.

[39] Dalquen DA, Anisimova M, Gonnet GH, Dessimoz C (2012). ALF - a simulation framework for genome evolution. Mol Biol Evol.

[40] Flicek P, Ahmed I, Amode MR, Barrell D, Beal K, Brent S, Carvalho-Silva D, Clapham P, Coates G, Fairley S, Fitzgerald S, Gil L, Garcia-Giron C, Gordon L, Hourlier T, Hunt S, Juettemann T, Kahari AK, Keenan S, Komorowska M, Kulesha E, Longden I, Maurel T, McLaren WM, Muffato M, Nag R, Overduin B, Pignatelli M, Pritchard B, Pritchard E, Riat HS, Ritchie GR, Ruffier M, Schuster M, Sheppard D, Sobral D, Taylor K, Thormann A, Trevanion S, White S, Wilder SP, Aken BL, Birney E, Cunningham F, Dunham I, Harrow J, Herrero J, Hubbard TJ, Johnson N, Kinsella R, Parker A, Spudich G, Yates A, Zadissa A, Searle SM (2013). Ensembl 2013. Nucleic Acids Res.

[41] Committee HGN. HUGO Gene Nomenclature Committee. http://www.genenames.org/. Accessed 12 Feb 2007.

[42] Wolfe KH, Shields DC (1997). Molecular evidence for an ancient duplication of the entire yeast genome. Nature.

[43] Dietrich FS, Voegeli S, Brachat S, Lerch A, Gates K, Steiner S, Mohr R, Pohlmann C, Luedi P, Choi SEA (2004). The Ashbya gossypii genome as a tool for mapping the ancient Saccharomyces cerevisiae genome. Science.

[44] Dujon B, Sherman D, Fischer G, Durrens P, Casaregola S, Lafontaine I, De Montigny J, Marck C, Neuveglise C, Talla E (2004). Genome evolution in yeasts. Nature.

[45] Sievers F, Wilm A, Dineen D, Gibson TJ, Karplus K, Li W, Lopez R, McWilliam H, Remmert M, Soding J, Thompson JD, Higgins DG (2011). Fast scalable generation of high quality protein multiple sequence alignments using Clustal Omega. Mol Syst Biol.

[46] Price MN, Dehal PS, Arkin AP (2009). FastTree: computing large minimum evolution trees with profiles instead of a distance matrix. Mol Biol Evol.

[47] Durand D, Halldorsson BV, Vernot B (2005). A hybrid micro-macroevolutionary approach to gene tree reconstruction. J Comput Biol.

[48] Goodman M, Czelusniak J, Moore GW, Romero-Herrera AE, Matsuda G (1979). Fitting the gene lineage into its species lineage a parsimony strategy illustrated by cladograms constructed from globin sequences. Syst Zool.

[49] Mahmudi O, Sjöstrand J, Sennblad B, Lagergren J (2013). Genome-wide probabilistic reconciliation analysis across vertebrates. BMC Bioinforma.

[50] Doyon JP, Chauve C, Hamel S (2009). Space of gene/species trees reconciliations and parsimonious models. J Comput Biol.

[51] Ali RH, Muhammad SA, Arvestad L. GenFamClust: an accurate, synteny-aware and reliable homology inference algorithm. figshare. 2015. doi:http://dx.doi.org/10.6084/m9.figshare.1536467.v4. 10.1186/s12862-016-0684-2 PMC4893229 27260514

