# Supplementary material for: GenFamClust: an accurate, synteny-aware and reliable homology inference algorithm
Source: BMC Evol Biol. 2016 Jun 4;16:120. doi: 10.1186/s12862-016-0684-2 (PMC4893229; doi:10.1186/s12862-016-0684-2)
Supplement: Additional file 1 — Additional documentation. Supplementary file containing additional documentation, source and processing of data sets, commands used to execute different software, and how the figures and tables were generated from raw data. (PDF 137 kb) [file 12862_2016_684_MOESM1_ESM.pdf]

Supplementary data – GenFamClust: an accurate, synteny-aware and reliable homology inference algorithm

Raja Hashim Ali[1], Sayyed Auwn Muhammad[1], Lars Arvestad[2,3,4],

1 KTH Royal Institute of Technology, Science for Life Laboratory, School of Computer Science and Communication, SE-17 177, Solna, Sweden.
2 Department of Numerical Analysis and Computer Science, Stockholm University, SE-100 44, Stockholm, Sweden.
3 Swedish e-Science Research Centre.
4 Science for Life Laboratory, SE-17 177, Solna, Sweden.

1 Data access and files

The complete raw and processed data, results and excel sheets underlying all figures and tables have been uploaded to figshare and are publicly available as zipped folder on the website `http://dx.doi.org/10.6084/m9.figshare.1536467`.

2 Datasets and Source explanation

The data, results and all data-sheets have been put in separate folders. The datasets are contained in ”Fungi Data.zip”, ”Multispecies Data.zip” and ”Simulated Data.zip”.

2.1 Simulated datasets

We generated size simulated datasets with varying gene similarity and gene order conservation. Simulated data was generated using Artificial Life Framework (ALF) and the settings for generatins simulations have been presented in the main manuscript and can also be viewed in ”used_alf-params.drw” files present in each dataset. For running ALF and how to generate data, please refer to the ALF manual distributed along with the software. The important characteristic in the dataset is the variation in gene order conservation and in the gene content. Fission, fusion, lateral gene transfer and functionalization events etc. are complications and their rates have been set to zero for these datasets. The gold standard for each dataset has been generated by using scripts and are present in each dataset folder in indexed (.bcl file) and with normal names (.txt file). We have also generated a mapping file (.map) for the gold standard where each sequence ID is mapped onto its index and the gene family ID it belongs to. The synteny file has also been generated by a script and is present as .hsf file. All other folders, e.g., DBancestral and VP, are generated by default during a simulation and are not useful in our analysis.

2.2 Fungi data

The Fungi data has been taken from Yeast Gene Order Browser (YGOB) ver. 7. It contains complete genomic information about twenty fungi species and can be found at `http://ygob.ucd.ie/ygob/data/v7-Aug2012`. The protein sequences for each species can be found from ”X_sequence.fsa” and the gene order information is present in ”X_genome.tab” file, where X is the name of the species. The synteny information was extracted from tab files and the sequence file headers were cleaned to remove extra information. This leads to the Fungi data synteny and sequence files present in this dataset.

The gold standard for this dataset has been given by YGOB, where they have clustered all genes and display each cluster as ”pillars” in ”pillars.tab” file from the website. The pillar file contains information about probable orthologs and ohnologs. It also hypothesizes about how the ancient chromosome would have looked in terms of gene order and also provides homology information for genomic regions that are not translated to proteins (e.g., trna, snRNA and Centrioles). We have removed the ancestral chromosome track and the pillars that contain untranslatable genomic regions leaving us with the pillars.tab file and the ”Removed_ancestral_track_and_RNA_pillars.tab” file given in the Gold Standard folder. We also have the indexed pillars file ”Index_2_Name_MapOfAllGenesInYGOBPillars.map” used in evaluation and comparison of YGOB with GFC.

2.3 Multispecies data

The multispecies dataset is a diverse dataset consisting of 17 Eukaryotic species. The human and mouse species are repeated as they are our query species and are also present in the reference data. The gold standard data taken from Song et al. paper consists of proteins from human and mouse, which means that the query data for this dataset consists of proteome from human and mouse. The reference data species were limited to 17 species and were chosen keeping two things in mind. First, we require species whose complete genome has been sequenced and whose genome is of satisfactorily good quality. Ensembl genome browser (`http://www.ensembl.org/index.html`) represented an excellent source for complete proteome with protein to gene mapping and generally well-assembled genomes. Secondly, from the list of available species in Ensembl, we chose model species in general since they have been thoroughly studied and are selected on evolutionarily and biologically solid grounds. The seventeen species in the reference dataset contain the proteome of human and mouse species and fifteen other species. The data and species have been downloaded from Ensembl version 72 and can be downloaded using biomart tool and the scientific name of the species.

One requirement for input to GenFamClust is that each protein in the proteome should be mapped to exactly one gene. While most proteins in most species in Ensembl database are mapped onto a single gene, some species like *Homo sapiens* and *Mus musculus* contain genes with more than one protein products (termed protein isoforms). We have selected the longest isoform by length as the protein representative of the gene to fulfill the GFC input requirement.

2.3.1 Gold standard dataset for metazoans

Table 1 displays the twenty gene families used as gold standard for metazoan dataset and displays the general properties of each family.

Table 1. Gene families in metazoan dataset and their general properties.

Gene Family	Members	Domain Architecture	Conservation Level
DVL	6	Cons MultiDomain	High
GATA	12	Cons MultiDomain	High
KIR	9	Cons MultiDomain	High
Notch	8	Cons MultiDomain	High
TRAF	12	Cons MultiDomain	High
ACSL	10	Single	High
FGF	44	Single	Average
FOX	78	Single	Average
Tbox	30	Single	Average
TNF	31	Single	Average
USP	76	Single	Low
WNT	38	Single	Low
ADAM	44	Variable MultiDomain	Average
Kinase	903	Variable MultiDomain	Low
Kinesin	56	Variable MultiDomain	Average
Laminin	22	Variable MultiDomain	Average
Myosin	45	Variable MultiDomain	Average
PDE	44	Variable MultiDomain	Average
SEMA	38	Variable MultiDomain	Average
TNFR	55	Variable MultiDomain	Average
All	1561	Diverse	Diverse
All-Kinase	658	Diverse	Diverse

Table showing gene family names, members and domain architecture of each family of test dataset. The families capture diversity in the size, domain architecture and level of sequence conservation within each family. The largest and most complex gene family, by far, is Kinases and to avoid biases in quality score, All-Kinase consists of all proteins except Kinase.

3 Input Files and syntax

Table 2 explains the input to a software and the format expected for the input. We changed all input into the corresponding format and then passed onto the different software. Note that some input files may not be required for one software but required for another (e.g., GFC explicitly differentiates between query files and reference files but other software do not!).

Table 2. Input files, syntax, and description

Filename	Description
PrepBlastfile.bl	Results from BLAST in ABC format (separated by tabs) where first two columns are gene names and third column is bitscore. For all software except NC, *PrepBlastfile* is tab delimited. For NC, *PrepBlastfile* should be space-delimited.
QuerySeq.fasta	File containing the query sequences only in fasta format. The headers of each sequence should match with the gene names in the *QuerySyn.hsf*.
QuerySyn.hsf	File containing the synteny or gene order information of query sequences in hsf format, i.e., tab-delimited species name, chromosome number, unique gene name and position on chromosome. The gene name should be unique and same as the one used as header in *QuerySeq.fasta* and it is assumed that the file has been sorted in ascending order on species name followed by chromosome followed by position on chromosome.
RefSeq.fasta	File containing the reference sequences only in fasta format. The headers of each sequence should match with the gene names in the *RefSyn.hsf*.
RefSyn.hsf	File containing the synteny or gene order information of reference sequences in hsf format, i.e., tab-delimited species name, chromosome number, unique gene name and position on chromosome. The gene name should be unique and same as the one used as header in *RefSeq.fasta* and it is assumed that the file has been sorted in ascending order on species name followed by chromosome followed by position on chromosome.
FASTAFILE	File containing all the sequences (*QuerySeq.fasta* + *RefSeq.fasta*) in fasta format.
PrepSeqFile.fasta	File containing all the prepared sequences (sequence headers are indexed according to "species index"."chr index"."gene index") in fasta format.
PrepQuerySeq.fasta	Query sequences in fasta format from *PrepSeqFile.fasta*
PrepRefSeq.fasta	Reference sequences in fasta format from *PrepSeqFile.fasta*

4 Commands to run software and scripts

Following are the list of homology inference software and the commands to run each software with the default values. Note that one can use the help menu to identify the available parameters and the default parameter values. We have however listed default value of each parameter in the data sheet "Default Parameter Settings.xlsx".

4.1 GFC-Single, GFC-Average and GFC-Complete

For convenience and brevity, we define the following command for GenFamClust,

```
GFC='java -Xmx2000m -Xms1800m -jar GenFamClust-1.0.0.jar'
```

which is used in the commands below.

4.1.1 Data preparator

```
$ GFC DataPreparator <QuerySeqs> <QuerySyn.hsf> -s <RefSeqs> -sy <RefSyn.hsf> -o
<PrepOutput>
```

QuerySeqs and RefSeqs are Fasta-files.

4.1.2 Data preparator

```
$ GFC BlastModule <PrepQuerySeq.fasta> -s <PrepRefSeq.fasta>
```

4.1.3 Compute NC score

```
$ NC_standalone -f <PrepBlastfile> > <NC_File.nc>
```

4.1.4 Compute synteny score

```
$ GFC SyntenyExtractor <QuerySyn.hsf> <NC_File.nc> -f <RefSyn.hsf> -o
<SynOutput.sysc>
```

4.1.5 Compute synteny correlation score

```
$ GFC SyntenyCorrelation <SynOutput.sysc> <QuerySyn.hsf> -o <SynCorr.syc>
```

4.1.6 Compute homology scores

```
$ GFC Evaluator <SynCorr.syc> -o <Homology.eva>
```

4.1.7 Compute gene families for Single linkage

```
$ GFC SparseClusterer <QuerySyn.hsf> <Homology.eva> -r <RefSyn.hsf> -m s -o
<ClusterResults.bcl>
```

4.1.8 Compute gene families for Average linkage

```
$ GFC SparseClusterer <QuerySyn.hsf> <Homology.eva> -r <RefSyn.hsf> -m a -o
<ClusterResults.bcl>
```

4.1.9 Compute gene families for Complete linkage

```
$ GFC SparseClusterer <QuerySyn.hsf> <Homology.eva> -r <RefSyn.hsf> -m c -o
<ClusterResults.bcl>
```

4.2 NC-Single

4.2.1 Compute NC score

```
$ NC_standalone -f <PrepBlastfile> > <NC_File.nc>
```

4.2.2 Compute gene families for Single linkage

```
$ GFC SparseClusterer <QuerySyn.hsf> <NC_File.nc> -r <RefSyn.hsf> -m s -o
<ClusterResults.bcl>
```

4.2.3 Compute gene families for Average linkage

```
$ GFC SparseClusterer <QuerySyn.hsf> <NC_File.nc> -r <RefSyn.hsf> -m a -o
<ClusterResults.bcl>
```

4.2.4 Compute gene families for Complete linkage

```
$ GFC SparseClusterer <QuerySyn.hsf> <NC_File.nc> -r <RefSyn.hsf> -m c -o
<ClusterResults.bcl>
```

4.2.5 Compute gene families for NC-Hierarchical

```
$ ./hcluster_sg <NC_File.nc> -o <NC_Hier.hcl>
```

4.3 hcluster_sg

```
$ ./hcluster_sg <PrepBlastFile.bl> -o <Result.hcl>
```

4.4 MCL

4.4.1 Extracting the e-value column

```
$ cut -f 1,2,11 <CompleteBlast.bl> > <EValueBlast.bl>
```

4.4.2 Applying constraints

```
$ mcxload -abc <EValueBlast.bl> --stream-mirror --stream-neg-log10 -stream-tf
'ceil(200)' -o <Result.mci> -write-tab <Result.tab>
```

4.4.3 Computing MCL

```
$ mcl <Result.mci> -I <inflation> -use-tab <Result.tab>
```

5 Results and data sheets underlying figures and tables

The results of all datasets and all figures are provided in the weblink and can be downloaded.

"Results for simulated data.zip" contains the final results for all methods and the cluster quality evaluation results for the software. Figure 3 in the main manuscript has been drawn using the results from this folder.

"Results for multispecies data.zip" contains the final results for all methods and the cluster quality, precision and recall evaluation results for the software. Figure 4, 5, 6 and 7, and Table 4 in the main manuscript are illustrations drawn from these results.

"Results for fungi data.zip" contains the final results for GFC and the comparisons with YGOB pillars. Figure 3, 8, and 9 and Table 5 in the main manuscript are illustrations drawn from these results.

"Data Sheets.zip" contain the final tables and results in excel data sheets from which various figures and tables in the main manuscript were generated.

5.1 Figure 2

The finalized concise results for Figure 2 can be found in Data sheet "Figure 2 – Fungi pillar gene distribution.xlsx".

5.2 Figure 3

The finalized concise results for Figure 3 can be found in Data sheet "Figure 3 – Simulated Data Results.xlsx".

5.3 Figure 4

The finalized concise results for Figure 4 can be found in Data sheet ”Figure 4 – ROC curve.xlsx”.

5.4 Figure 5

The finalized concise results for Figure 5 can be found in Data sheet ”Figure 5 – Accuracy of GFC vs others on multispecies.xlsx” and the raw results for figure in ”Intermediate file for Figure 5.xlsx”.

5.5 Figure 7 and Figure 8

The finalized concise results for Figure 7 and Figure 8 can be found in Data sheet ”Figure 6 and 7 -Comparison of Proteom Clusterings.xlsx”.

5.6 Figure 9

The finalized concise results for Figure 9 can be found in Data sheet ”Figure 8 – GFC vs YGOB pillars.xlsx”.

5.7 Table 3 and 4

The finalized concise results for Figure 2 can be found in Data sheet ”Table 3 and 4 Data – MultiSpecies and agreement between methods.xlsx”.
